# Deregulation of purine pathway in *Bacillus subtilis* and its use in riboflavin biosynthesis

**DOI:** 10.1186/s12934-014-0101-8

**Published:** 2014-07-15

**Authors:** Ting Shi, Yongcheng Wang, Zhiwen Wang, Guanglu Wang, Dingyu Liu, Jing Fu, Tao Chen, Xueming Zhao

**Affiliations:** 1 Department of Biochemical Engineering, School of Chemical Engineering and Technology, Tianjin University, Tianjin, People’s Republic of China; 2 Key Laboratory of Systems Bioengineering, Ministry of Education, Tianjin University, Tianjin, People’s Republic of China; 3 Collaborative Innovation Center of Chemical Science and Engineering (Tianjin), Tianjin University, Tianjin, People’s Republic of China; 4 Edinburgh-Tianjin Joint Research Centre for Systems Biology and Synthetic Biology, Tianjin University, Tianjin, People’s Republic of China

**Keywords:** Bacillus subtilis, Purine pathway, Transcription repression, Feedback inhibition, Deregulation, Riboflavin

## Abstract

**Background:**

Purine nucleotides are essential metabolites for living organisms because they are involved in many important processes, such as nucleic acid synthesis, energy supply, and biosynthesis of several amino acids and riboflavin. Owing to the pivotal roles of purines in cell physiology, the pool of intracellular purine nucleotides must be maintained under strict control, and hence the *de novo* purine biosynthetic pathway is tightly regulated by transcription repression and inhibition mechanism. Deregulation of purine pathway is essential for this pathway engineering in *Bacillus subtilis*.

**Results:**

Deregulation of purine pathway was attempted to improve purine nucleotides supply, based on a riboflavin producer *B. subtilis* strain with modification of its *rib* operon. To eliminate transcription repression, the *pur* operon repressor PurR and the 5’-UTR of *pur* operon containing a guanine-sensing riboswitch were disrupted. Quantitative RT-PCR analysis revealed that the relative transcription levels of purine genes were up-regulated about 380 times. Furthermore, site-directed mutagenesis was successfully introduced into PRPP amidotransferase (encoded by *purF*) to remove feedback inhibition by homologous alignment and analysis. Overexpression of the novel mutant PurF (D293V, K316Q and S400W) significantly increased PRPP amidotransferase activity and triggered a strong refractory effect on purine nucleotides mediated inhibition. Intracellular metabolite target analysis indicated that the purine nucleotides supply in engineered strains was facilitated by a stepwise gene-targeted deregulation. With these genetic manipulations, we managed to enhance the metabolic flow through purine pathway and consequently increased riboflavin production 3-fold (826.52 mg/L) in the *purF-VQW* mutant strain.

**Conclusions:**

A sequential optimization strategy was applied to deregulate the *rib* operon and purine pathway of *B. subtilis* to create genetic diversities and to improve riboflavin production. Based on the deregulation of purine pathway at transcription and metabolic levels, an extended application is recommended for the yield of products, like inosine, guanosine, adenosine and folate which are directly stemming from purine pathway in *B. subtilis*.

## Background

Purine nucleotides are essential metabolites for cellular physiology because they are structural components of both DNA and RNA, energy carriers (i.e. ATP and GTP) and enzyme cofactors (i.e. NAD^+^ and NADP^+^). In addition, purine nucleotides are involved in many important anabolic pathways for biosynthesis of several amino acids and vitamins such as folic acid and riboflavin, which are of significant economic interest for the biotechnology industry [[1]-[3]].

The *de novo* purine biosynthetic pathway is nearly ubiquitous and leads the conversion of PRPP and glutamine into IMP through 10 different enzymatic reactions, after which IMP can be transformed into AMP or GMP in two additional steps. Alternatively, purines can also be transformed directly to their nucleoside monophosphate derivatives using the intracellular pool of PRPP for recycling of nucleobases through the salvage pathways [[4]]. Owing to the pivotal roles of purines in cell physiology, the pool of intracellular purine nucleotides must be maintained under strict control, and hence the *de novo* purine biosynthetic pathway is tightly regulated by transcription repression and inhibition mechanism [[5]].

**Figure 1:**
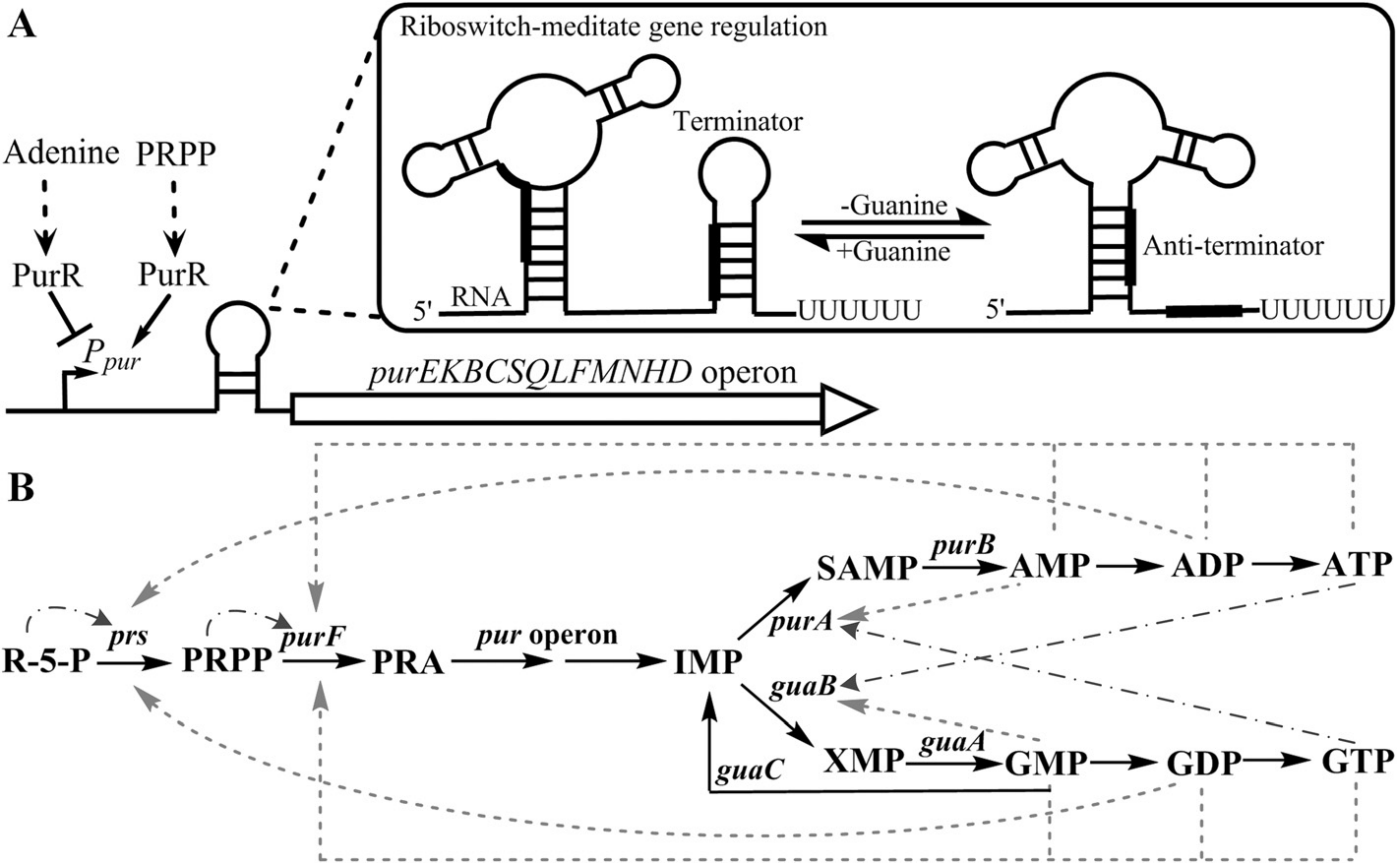
**Regulation of the*de novo*purine biosynthetic pathway in*B. subtilis.*(A)** Transcription regulation of *pur* operon by PurR and a guanine-sensing riboswitch. The *pur* operon repressor PurR is the main regulator of *pur* operon. PRPP antagonizes DNA binding of PurR, whereas abundant adenine enhances the activity of PurR to repress the expression of *pur* operon*.* The FMN riboswitch employs a mechanism for gene control that relies on the mutually exclusive formation of intrinsic transcription terminator and antiterminator stem structures. **(B)** Feedback inhibition regulation of purine pathway at metabolic levels. The black solid lines represent metabolic conversions, the dash-dotted lines depict enzyme activation, and the dotted lines depict feedback inhibition. *Abbreviations*: R5P, ribose-5-phosphate; PRPP, 5-phospho-α-D-ribosyl-1-pyrophosphate; PRA, 5-phospho-α-D-ribosylamin; IMP, inosine 5’-mono-phosphate; XMP, xanthosine 5’-mono-phosphate; GMP, guanosine 5’-mono-phosphate; GDP, guanosine 5’-di-phosphate; GTP, guanosine 5’-tri-phosphate; SAMP, succinyladenosine mono-phosphate; AMP, adenosine 5’-mono-phosphate; ADP, adenosine 5’-di-phosphate; ATP, adenosine 5’-tri-phosphate; *prs*, PRPP synthetase; *purF*, PRPP amidotransferases; *purA*, adenylosuccinate synthetase; *purB*, adenylosuccinate lyase; *guaB*, IMP dehydrogenase; *guaA*, GMP synthase; *guaC*, GMP reductase.

In *B. subtilis*, most of purine genes for AMP and GMP synthesis are negatively regulated at transcriptional level [[6]] (Figure 1A). The purine repressor PurR, encoded by *purR*, and specific DNA sequences in the upstream control regions of affected genes, PurBoxes, are essential elements to repress transcription from a number of genes with functions in the synthesis, transport, and metabolism of purines [[7]]. The PurR-PurBox system regulates the transcription of all genes encoding enzymes for synthesis of IMP, AMP and GMP from PRPP. And this system also regulates transcription of the *purR* operon (*purR* and *yabJ*) and of several genes involved in cofactor biosynthesis (*glyA* and *folD*), purine salvage (*guaC* and *xpt*), and purine transport (*pbuG*, *pbuO* and *pbuX*) [[8]]. The effectors acting on *purR* expression vary among species. For example, hypoxanthine and guanine are co-repressors of PurR in *Escherichia coli* [[9]], while PRPP antagonizes DNA binding of PurR and abundant adenine represses PRPP synthesis in *B. subtilis* [[10]]. The activity of PurR can be enhanced to repress the expression of target genes when purine nucleotides become available from the environment [[11]]. Moreover, the transcription of *pur* operon in *B. subtilis* is further negatively regulated by a guanine-sensing riboswitch. The 5’-UTR of *pur* operon mRNA contains a stretch so called “riboswitch”, which binds to guanine to form a specific fold to enable a terminator structure to abort the transcription prematurely [[12]]. Disruption of *purR* gene and the guanine-sensing riboswitch in *B. subtilis* increased the activity of *pur* operon promoter so as to enhance purine nucleotides biosynthesis [[13]].

Regarding feedback inhibition mechanism, enzyme activities are subjected to end product-feedback inhibition in purine pathway of *B. subtilis*, e.g. PRPP synthetase, PRPP amidotransferase, adenylsuccinate synthetase and IMP dehydrogenase [[5],[14]] (Figure 1B). PRPP amidotransferase, encoded by *purF*, catalyzes the initial reaction of purine pathway and is the key regulatory enzyme in this pathway. Expression of *purF* is repressed at transcriptional level by extracellular purines [[13]], and the activity of PRPP amidotransferase is rigorously regulated by feedback inhibition through the specific binding of adenine and guanine nucleotides [[5]]. As reported, the structural features of *B. subtilis* PRPP amidotransferase have been described, and several mutated residues (S283A, K305Q, R307Q and S347A) were successfully introduced into PRPP amidotransferase to release it from feedback regulation [[15]]. The inhibitory properties of PRPP amidotransferase in other strains such as *E. coli* and *Ashbya gossypii* were also abolished by site-directed mutagenesis [[16]-[18]]. For example, overexpression of the desensitized PurF (K326Q and P410W) significantly redirected carbon flow through the *de novo* purine biosynthetic pathway in inosine-producing strain *E. coli* W3110 [[19]]. Jiménez et al [[18]] had introduced residue mutations (D310V, K333Q and A417W) into PRPP amidotransferase (encoded by Ag*ADE4*) in *A. gossypii*, and the mutated Ag*ADE4* was overexpressed to abolish the adenine-mediated transcription repression and to reduce sensitivity against inhibition by ATP and GTP. Similarly, new residue mutations of *B. subtilis* PRPP amidotransferase are explored to release feedback inhibition and to direct the metabolic flux of purine pathway, which are of importance for the yield of products directly stemming from purine pathway in *B. subtilis*.

As mentioned above, the purine biosynthetic network is closely connected to many important metabolic pathways for biotechnology application. *B. subtilis* has a long tradition as safe and stable producer for purine nucleotides [[13],[20],[21]]. It should be noted that *B. subtilis* has constituted a paradigm of environmentally friendly “white” biotechnology with regard to industrial riboflavin overproduction [[22],[23]]. Riboflavin is an important extensive research compound, since it serves as a precursor of flavin mononucleotide (FMN) and flavin adenine dinucleotide (FAD), and is also an essential nutrient for humans and animals as food additive [[1]]. For biosynthesis of riboflavin in *B. subtilis*, poor availability of purine nucleotides may limit the precursor GTP supply. Genetic manipulation of the *de novo* purine biosynthetic pathway in *B. subtilis* could considerably enhance the production of riboflavin.

In this work, we tried to engineer the *rib* operon in a riboflavin producer *B. subtilis* strain to direct necessary high levels of carbon flow through the riboflavin biosynthetic pathway, including overexpression of the key gene (*ribA*), substitution the native promoter *ribP*1 with strong promoter *P*_43_ and disruption of the RFN regulatory region *ribO*. Afterwards, a sequential optimization strategy was applied to deregulate purine pathway in the *rib* operon manipulated *B. subtilis* mutant strain. The repressor PurR and the 5’-UTR of *pur* operon containing a guanine-sensing riboswitch were both disrupted to eliminate transcription repression. And quantitative RT-PCR analysis revealed that the relative transcription levels of purine genes were up-regulated. Site-directed mutagenesis was subsequently introduced into PRPP amidotransferase to remove feedback inhibition by homologous alignment and analysis. The characteristics of mutant PRPP amidotransferase were analyzed, and intracellular metabolite profile changes of different engineered strains confirmed significant deregulation of purine pathway. With these manipulations, we managed to enhance the metabolic flow through purine pathway and consequently increased riboflavin production 3-fold (826.52 mg/L) in the *purF-VQW* mutant strain.

## Results

### Genetic manipulation of the *B. subtilis rib* operon for riboflavin production

In order to direct the necessary high levels of carbon flow through the riboflavin biosynthetic pathway in *B. subtilis*, we attempted to engineer the *rib* operon in riboflavin producer strain BS77 that contained mutations in regulatory gene *ribC* and RFN regulatory element *ribO* (unpublished work). The strong constitutive promoter *P*_43_ derived from *B. subtilis* 168 was firstly inserted into upstream of *ribA* in BS77 to generate mutant strain BS89. Subsequently, the *rib* operon was constitutively overexpressed in mutant strain BS89 by either replacing the native promoter *ribP1* and *ribO* with strong promoter *P*_43_ or by deleting *ribO*, resulting in mutant strains BS93 and BS96, respectively.

**Figure 2:**
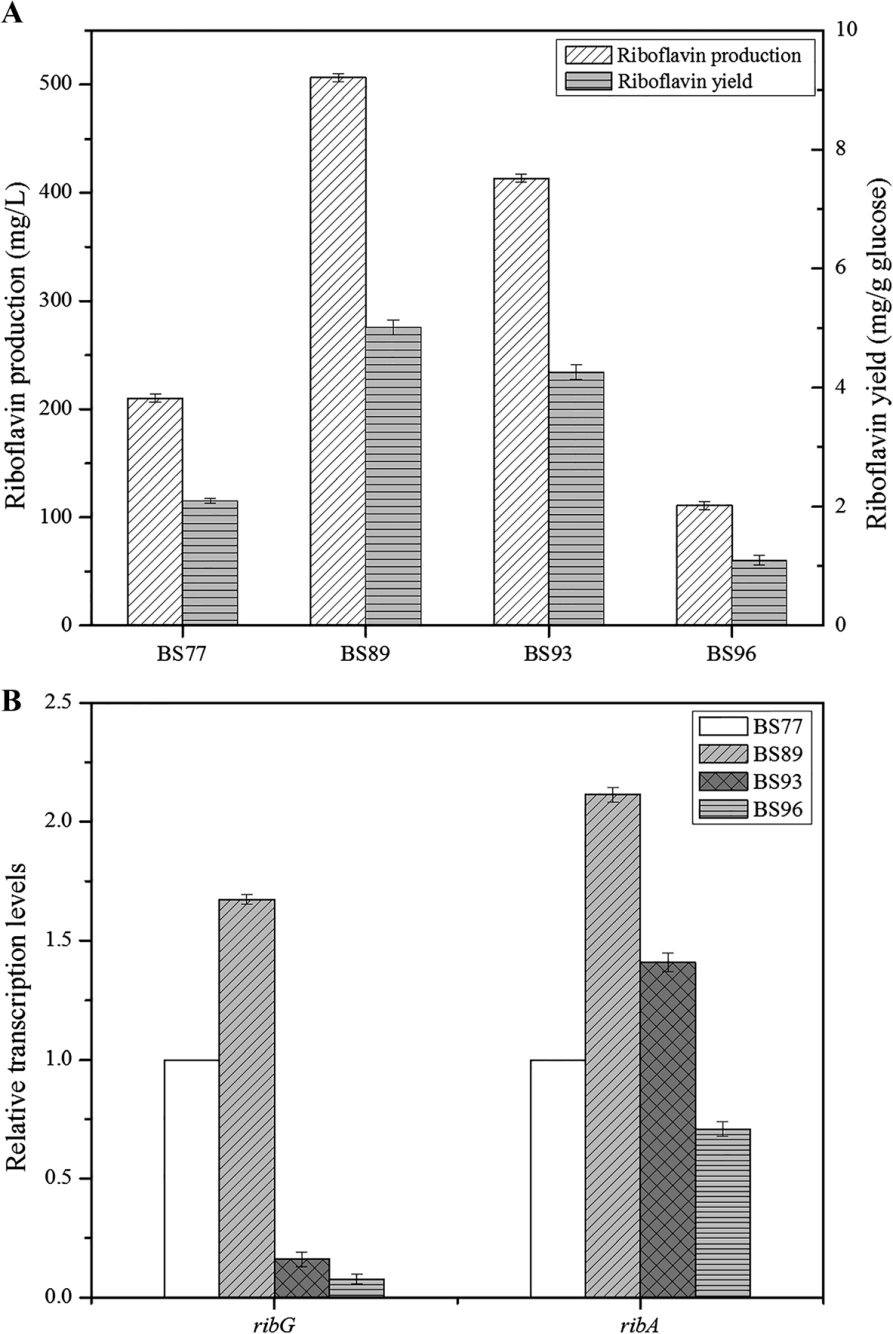
**Influence of various manipulations of*B. subtilis rib*operon on riboflavin production and genes expression. (A)** Comparison of riboflavin production and yield between genetically modified *rib* operon mutant strains developed in this study. **(B)** Changes of the relative transcription levels of *rib* genes (*ribG* and *ribA*) between mutant strains. Results were averages from three independent experiments, and error bars represented the standard errors of the means.

These mutant strains were compared with respect to physiological parameters and riboflavin production to investigate the effects of different genetic modifications (Table 1, Figure 2A). Overexpression of *ribA* in mutant BS89 led to approximately 1.4 times increase of riboflavin production from 210.25 ± 3.73 mg/L to 506.45 ± 3.50 mg/L, coupling with significant enhancement of the specific riboflavin production rate from 32.85 ± 2.66 μmol/g CDW/h to 41.65 ± 1.52 μmol/g CDW/h. However, constitutive expression of riboflavin biosynthesis pathway showed contrary results. Replacement of the native promoter *ribP*1 and *ribO* with strong promoter *P*_43_ in mutant BS93 resulted in riboflavin production decrease from 506.45 ± 3.50 mg/L to 341.87 ± 7.85 mg/L. Only *ribO* disruption in BS96 led the riboflavin production decreased to 111.09 ± 3.73 mg/L. The specific riboflavin production rates of BS93 and BS96 were decreased from 41.65 ± 1.52 to 30.80 ± 2.72 and 6.52 ± 1.79 μmol/g CDW/h, respectively.

**Table 1:** **Metabolic characterizations of** ***B. subtilis*** **strains in minimal medium supplemented with 20 g/L glucose**

**Strains**	**BS77**	**BS89**	**BS93**	**BS96**	**BS102**	**BS103**	**BS104**	**BS106**	**BS107**	**BS110**	**BS111**
Specific growth rate (h^-1^)	0.66 ± 0.01	0.64 ± 0.00	0.59 ± 0.01	0.62 ± 0.00	0.61 ± 0.01	0.61 ± 0.01	0.56 ± 0.01	0.60 ± 0.01	0.55 ± 0.01	0.45 ± 0.01	0.64 ± 0.04
Specific riboflavin production rate (μmol•g^-1^CDW•h^-1^)	32.85 ± 2.66	41.65 ± 1.51	30.80 ± 2.72	6.52 ± 1.79	32.74 ± 1.81	35.10 ± 1.21	34.96 ± 1.58	51.46 ± 3.58	27.15 ± 2.04	32.79 ± 0.48	61.15 ± 0.85
Specific glucose uptake rate (mmol•g^-1^CDW•h^-1^)	18.82 ± 2.08	16.25 ± 0.96	17.02 ± 1.02	19.45 ± 1.54	16.13 ± 0.13	17.35 ± 1.06	14.33 ± 0.26	15.38 ± 0.39	12.35 ± 0.54	8.69 ± 0.19	18.94 ± 3.00
Riboflavin yield (mg/g glucose)	2.10 ± 0.02	5.02 ± 0.06	4.26 ± 0.20	1.10 ± 0.04	5.21 ± 0.07	4.97 ± 0.14	5.56 ± 0.45	6.55 ± 0.50	5.12 ± 0.05	8.46 ± 0.30	5.84 ± 0.04

Bacteria were cultivated with 50 mL minimal medium in a 500 mL flask shaking at 240 rpm and 41°C; data were the average values of three parallel experiments.

The relative transcription levels of *rib* genes (*ribG* and *ribA*) were measured in different mutant strains by quantitative RT-PCR (Figure 2B). The relative transcription levels of *ribG* and *ribA* in mutant BS89 were significantly enhanced by approximately 2-fold higher than that of BS77. These results convincingly demonstrated that *ribA* overexpression could raise the metabolic flux of rate-limiting step, reflecting its direct contribution on riboflavin overproducing phenotype. Whereas the relative transcription levels of *ribG* and *ribA* in BS93 and BS96, constitutive expression of *rib* operon based on BS89, were decreased drastically, indicating that *ribO* disruption was probably the main reason for riboflavin yield drawdown. Therefore, mutant BS89 was used as the original strain to be manipulated for deregulating the *de novo* purine biosynthetic pathway through comprehensive analysis.

### Deregulation of purine pathway for enhancement of transcription levels

A sequential deregulation strategy was applied to elevate the transcription levels of purine pathway. The *purR* gene was disrupted to eliminate the transcription repression on purine pathway. Meanwhile, the original -10 sequence (TAAGAT) of *Ppur* (the *pur* operon promoter region) and the 5’-UTR of *pur* operon containing a guanine-sensing riboswitch were both engineered to increase the *pur* operon promoter activity.

**Figure 3:**
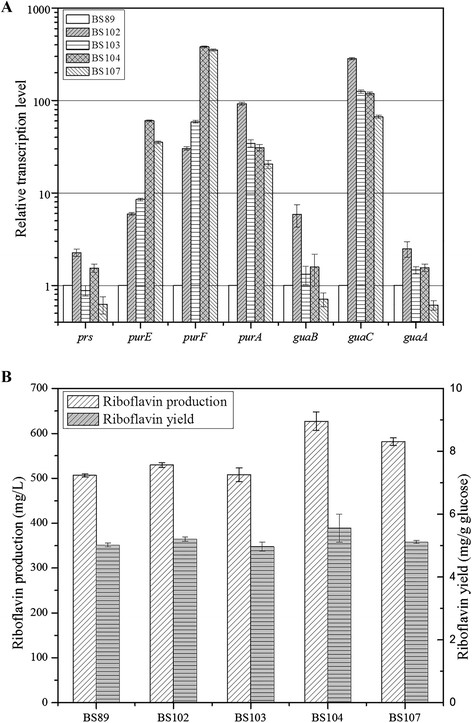
**Deregulation of purine pathway for enhancement of the relative transcription levels and riboflavin production.(A)** Changes of the relative transcription levels of purine genes between different deregulated strains developed in this study. **(B)** Comparison of riboflavin production and yield between different deregulated strains. Results were averages from three independent experiments, and error bars represented the standard errors of the means.

The first target was *purR* gene, encoding *pur* operon repressor PurR, which was the main regulator of both *pur* operon and other genes related to the biosynthesis, transport, and metabolism of purines in *B. subtilis.* Disruption of *purR* gene in BS89 generated mutant strain BS102. The effect was evaluated with regard to transcription levels in BS102. Figure 3A showed *purR* deletion elevated all the relative transcription levels of seven key purine genes involved in purine metabolism. Remarkably, the relative transcription levels of *purA* and *guaC* were both up-regulated about 100 times higher than BS89, and the relative transcription levels of *purE* and *purF* were up to 30 times higher, revealing its effective deregulation on purine pathway. As for riboflavin production, mutant strain BS102 produced 529.54 ± 5.29 mg/L riboflavin with yield of 5.21 ± 0.07 mg/g glucose, representing 6% increased production comparing with BS89 (506.45 ± 3.50 mg/L) (Figure 3B).

For improving the *pur* operon promoter activity, the original -10 sequence (TAAGAT) of *Ppur* in BS102 was mutated with frequently used -10 sequence (TATAAT) in *B. subtilis* [[13]] to generate mutant strain BS103 (-10*). A 75-bp region which was defined as *att* corresponding to the attenuator region in 5’-UTP [[12]] was also deleted, leading to mutant strains BS104 (Δ*att*) based on BS102 and BS107 (-10*, Δ*att*) based on BS103, respectively. In comparison with the riboflavin production of 529.54 ± 5.29 mg/L in BS102, it was drastically enhanced to 627.15 ± 20.61 mg/L in Δ*att* mutant strain BS104, and less enhanced to 581.46 ± 8.34 mg/L in the combination of -10* and Δ*att* mutant strain BS107, but almost unchanged in -10* mutant strain BS103 (507.74 ± 15.33 mg/L) (Figure 3B). Noticeably, from the point of relative transcription levels, the *att* deletion was also efficacious for *pur* operon deregulation. As shown in Figure 3A, it presented the highest relative transcription levels of *purE* and *purF* than other mutant strains. Meanwhile, the relative transcription levels of *guaC* and *purA* were declined to some extent by this deregulation manipulation of *att* deletion, which was in favor of the accumulation of riboflavin. Physiological parameters and riboflavin production indicated *att* deletion played a vital role in improving the *pur* operon promoter activity, while the -10* point mutation failed to take effect.

### Identification of the residue mutations for PurF to release PRPP amidotransferase from feedback regulation

**Figure 4:**
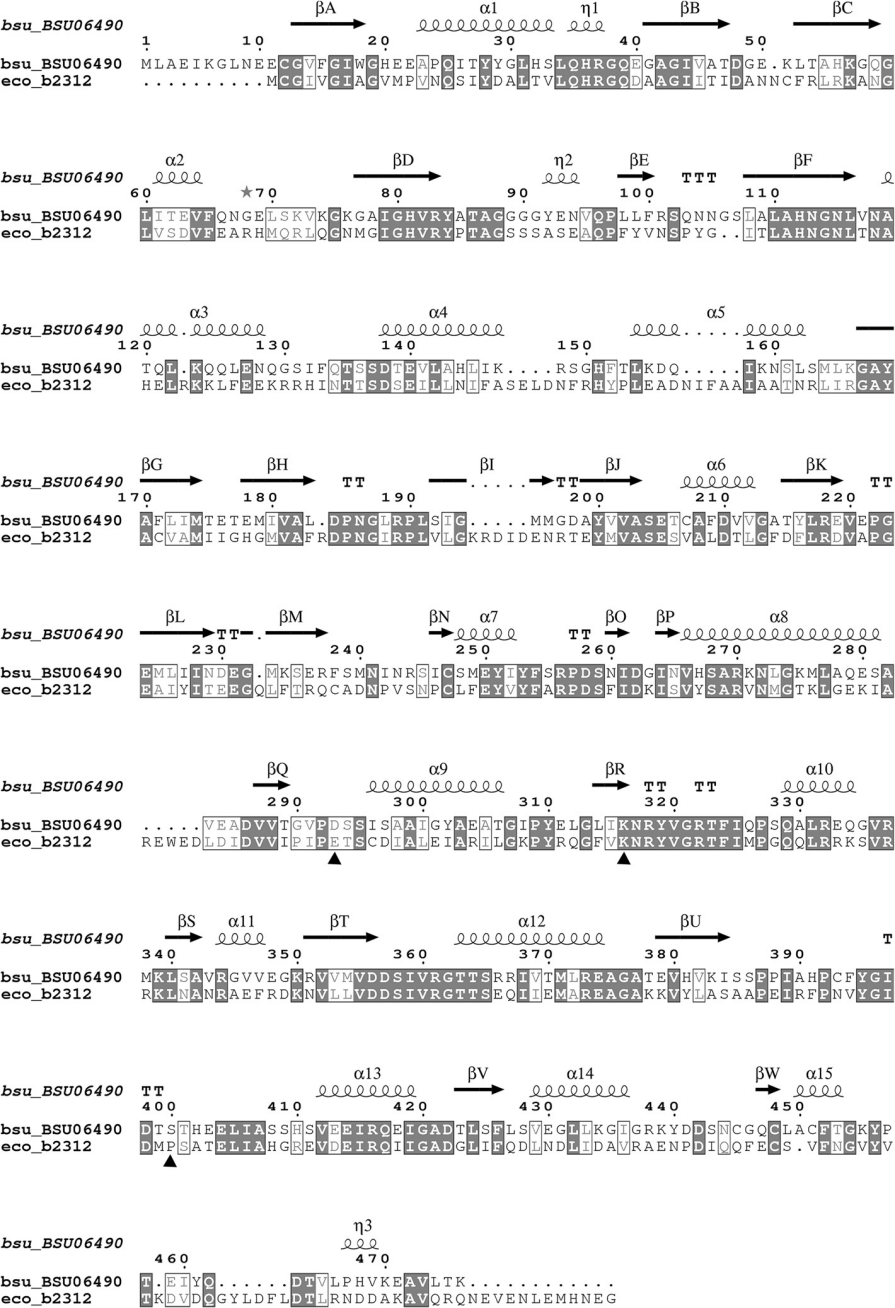
**Alignment of the amino acid sequences of*E. coli*and*B. subtilis*PRPP amidotransferases.** The amino acids were shown in single-letter code. Identical residues were shaded and conserved residues were in box. Symbols α and β represented the secondary structure elements for two proteins. The amino acid residues in the box referred to the conservative replacement of amino acid residues. The alignment was performed with the program ESPript 3.0. Residues replaced by site-directed mutagenesis were marked with a closed triangle. *Bsu*_BSU06490, *B. subtilis* PRPP amidotransferases; *eco*_b2312, *E. coli* PRPP amidotransferases.

PRPP amidotransferases activity is regulated by feedback inhibition through the specific binding of purine nucleotides to certain previously determined residues [[5],[24]]. Here, a multiple sequence alignment was applied to compare the amino acid sequences of *E. coli* and *B. subtilis* PRPP amidotransferases with the program ESPript 3.0. This alignment indicated that PurF in *B. subtilis* shared 36.56% amino acid identity with *E. coli* (Figure 4). Amino acid residues E304, K326 and P410, identified in *E. coli* PurF as being involved in allosteric regulation, were conserved in *B. subtilis* PurF. Thus, the known feedback-resistant mutations in *E. coli* PurF, glutamic acid to valine at amino acid residue 304 (E304V), lysine to glutamine at amino acid residue 326 (K326Q), and proline to tryptophan at amino acid residue 410 (P410W), were supposed to correspond to the D293V, K316Q and S400W mutations in *B. subtilis* PurF (Figure 4). As we speculated, aspartic acid^293^ was changed to valine (V), removing the carboxyl group that might be important for GTP binding; lysine^316^ was replaced by a glutamine residue (Q) to eliminate the amino group that probably interacts with GTP; and serine^400^ was changed to tryptophan (W), presumably causing a rearrangement of protein structure due to the introduction of bulky aromatic group of tryptophan and significantly attenuating the inhibitory effect of ATP.

To deregulate the committed step encoded by *purF* in purine pathway, a series of mutants were constructed. The wild-type *purF* in BS104 was firstly overexpressed, via inserting a strong promoter *P*_43_ upstream of *purF*, to generate mutant strain BS106. Then, the three residue mutations (D293V, K316Q and S400W) were introduced into BS106 to yield mutant strain BS110 with *purF-VQW* mutation module. Besides, mutated *purF* gene from *E. coli* containing mutations K326Q and P410W was successfully introduced into strain BS106 using plasmid pSS-purF*(eco)-FB to replace the wild-type *purF*, which was named BS111.

### Influence of *purF* mutant expression on riboflavin production and metabolic characteristics

**Figure 5:**
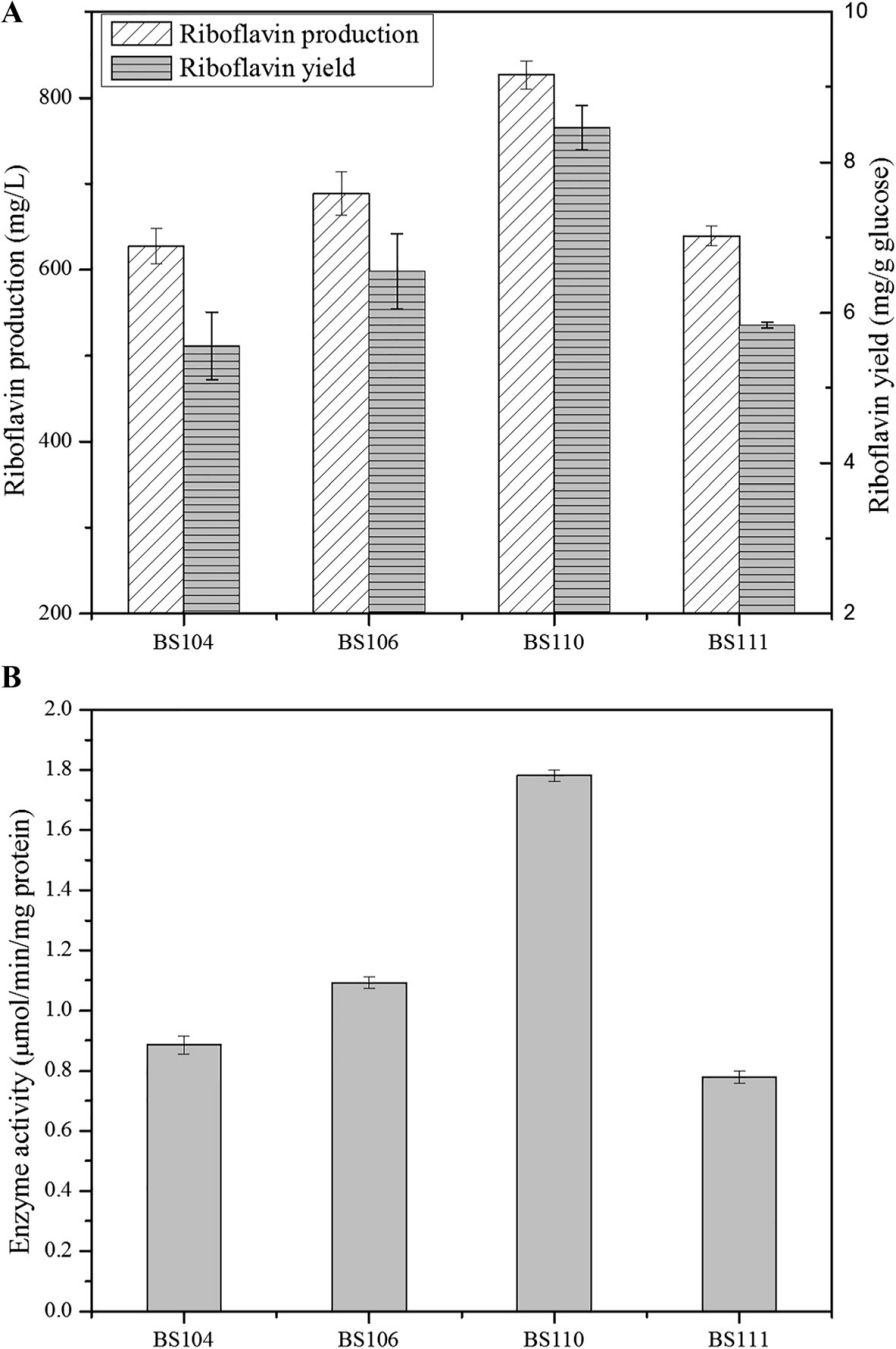
**Influence of*purF*mutant expression on riboflavin production and enzyme activities.(A)** Comparison of riboflavin production and yield between different *purF* manipulated mutant strains. **(B)** Changes of PRPP amidotransferases activities of different *purF* manipulated mutant strains. Results were averages from three independent experiments, and error bars represented the standard errors of the means.

To determine the effects of different markerless mutations of *purF*, we quantified physiological parameters and riboflavin production in mutant strains. As shown in Figure 5A and Table 1, overexpression of *purF* in strain BS106 led to approximately 10% improvement in riboflavin production than that of BS104 (from 627.15 ± 20.61 mg/L to 688.42 ± 25.57 mg/L), coupling with significant enhancement of the specific riboflavin production rate from 34.96 ± 1.58 μmol•g^-1^CDW•h^-1^ to 51.46 ± 3.58 μmol•g^-1^CDW•h^-1^. And the specific growth rate and the specific glucose uptake rate were both slightly improved. In addition, overexpression mutated *purF* gene from *E. coli* under strong promoter *P*_43_ in strain BS111 did not increase riboflavin production, even dropped slightly (from 688.42 ± 25.57 mg/L to 639.10 ± 1.56 mg/L).

Strain BS110 with *purF-VQW* mutation showed obvious riboflavin overproduction, which had almost 20% enhancement from 627.15 ± 20.61 mg/L to 826.52 ± 6.27 mg/L, and 29% increase of yield from 6.55 ± 0.50 mg/g glucose to 8.46 ± 0.30 mg/g glucose compared with BS106. Nevertheless, the specific riboflavin production rate in BS110 was decreased from 51.46 ± 3.58 μmol•g^-1^CDW•h^-1^ to 32.79 ± 0.48 μmol•g^-1^CDW•h^-1^, although it exhibited the highest riboflavin yield. At the same time, the specific growth rate was also decreased significantly (Table 1). It was probably due to the prolonged fermentation time of strain BS110 (from 72 h to 96 h). Combining with the intracellular metabolite target analysis (Table 2), it suggested that inserting *purF-VQW* mutation enhanced the concentrations of GMP, GDP and GTP compared with BS106, which was in favor of riboflavin production. But the concentrations of AMP, ADP and ATP decreased drastically, thus affecting the specific growth rate and the specific riboflavin production rate, as ATP was the direct source of energy for maintenance of cell metabolism. However, a defective GMP reductase (*guaC*) in BS110 was found to reduce conversion of GMP to IMP, and the specific growth rate of *guaC* disruption mutant strain was restored to some extent.

**Table 2:** **Concentrations of purine intermediates [μmol g CDW**^**-1**^**] determined for** ***B. subtilis*** **mutants**

**Strains**	**Concentration [μmol g CDW**^**-1**^**]**							
	BS89	BS102	BS103	BS104	BS106	BS107	BS110	BS111
PRPP	0.032 ± 0.006	0.042 ± 0.003	0.054 ± 0.007	0.054 ± 0.009	0.04 ± 0.011	0.066 ± 0.017	0.066 ± 0.013	0.024 ± 0.001
IMP	1.13 ± 0.12	3.62 ± 0.22	2.53 ± 0.24	3.58 ± 0.43	3.74 ± 0.58	4.31 ± 0.34	3.79 ± 0.27	2.26 ± 0.28
AMP	14.36 ± 2.13	32.45 ± 3.18	15.35 ± 1.25	29.82 ± 3.12	48.12 ± 3.53	47.96 ± 3.19	25.00 ± 3.01	4.80 ± 0.29
ADP	0.15 ± 0.011	0.16 ± 0.013	0.079 ± 0.005	0.14 ± 0.017	0.24 ± 0.031	0.26 ± 0.018	0.12 ± 0.017	0.024 ± 0.019
ATP	0.02 ± 0.002	0.027 ± 0.007	0.028 ± 0.005	0.011 ± 0.009	0.021 ± 0.003	0.04 ± 0.007	0.011 ± 0.003	0.004 ± 0.002
GMP	2.37 ± 0.16	9.25 ± 1.11	4.20 ± 0.55	9.36 ± 0.87	4.63 ± 0.28	1.80 ± 0.13	9.42 ± 1.12	3.45 ± 0.35
GDP	0.17 ± 0.012	0.19 ± 0.011	0.14 ± 0.005	0.41 ± 0.032	0.22 ± 0.019	0.42 ± 0.035	0.35 ± 0.043	0.13 ± 0.015
GTP	0.044 ± 0.012	0.056 ± 0.016	0.039 ± 0.009	0.067 ± 0.011	0.046 ± 0.014	0.047 ± 0.009	0.062 ± 0.017	0.024 ± 0.012

### Engineered forms of PRPP amidotransferase were metabolically deregulated

With respect to enzyme activity, significant changes for PRPP amidotransferase were observed in different mutant strains including BS104, BS106, BS110 and BS111. As shown in Figure 5B, the enzyme activity in BS106 was increased by 23% compared to the control strain BS104, indicating that overexpression of *purF* gene under strong promoter *P*_43_ was an effective means for improving the PRPP amidotransferase activity. Moreover, significant enhancement of PRPP amidotransferase activity in BS110 containing *purF*-*VQW* was observed, which was 1.63 times higher than that of BS106 and 2 times higher than that of BS104. It could be explained that residue modifications in the active centers frequently resulted in a drastic alteration in enzyme characteristics. To our surprise, substitution of the *B. subtilis purF* gene by the mutated *purF* gene from *E. coli* in BS111 failed to enhance enzyme activity and, on the contrary, the enzyme activity had a slight reduction. In the present work, the same promoter *P*_*43*_ and ribosome binding site were used to control the expression of *purF* or its derivations derived from *B. subtilis* or *E. coli*. So the different phenotypes of mutant *purF* should not be attributed to the control elements of promoter or ribosome binding site for gene expression. Further investigation was carried out to test whether there were rare codons existed in mutant *purF* from *E. coli.* The results exhibited that 21 rare codons were used less than 10% for heterologous expression in *B. subtilis.* Rare codons can reduce the efficiency of translation or even disengage the translational machinery [[25]-[28]]. Taking the above mentioned into consideration, the low expression of mutant *purF* derived from *E. coli* failed to enhance the enzyme activity.

**Figure 6:**
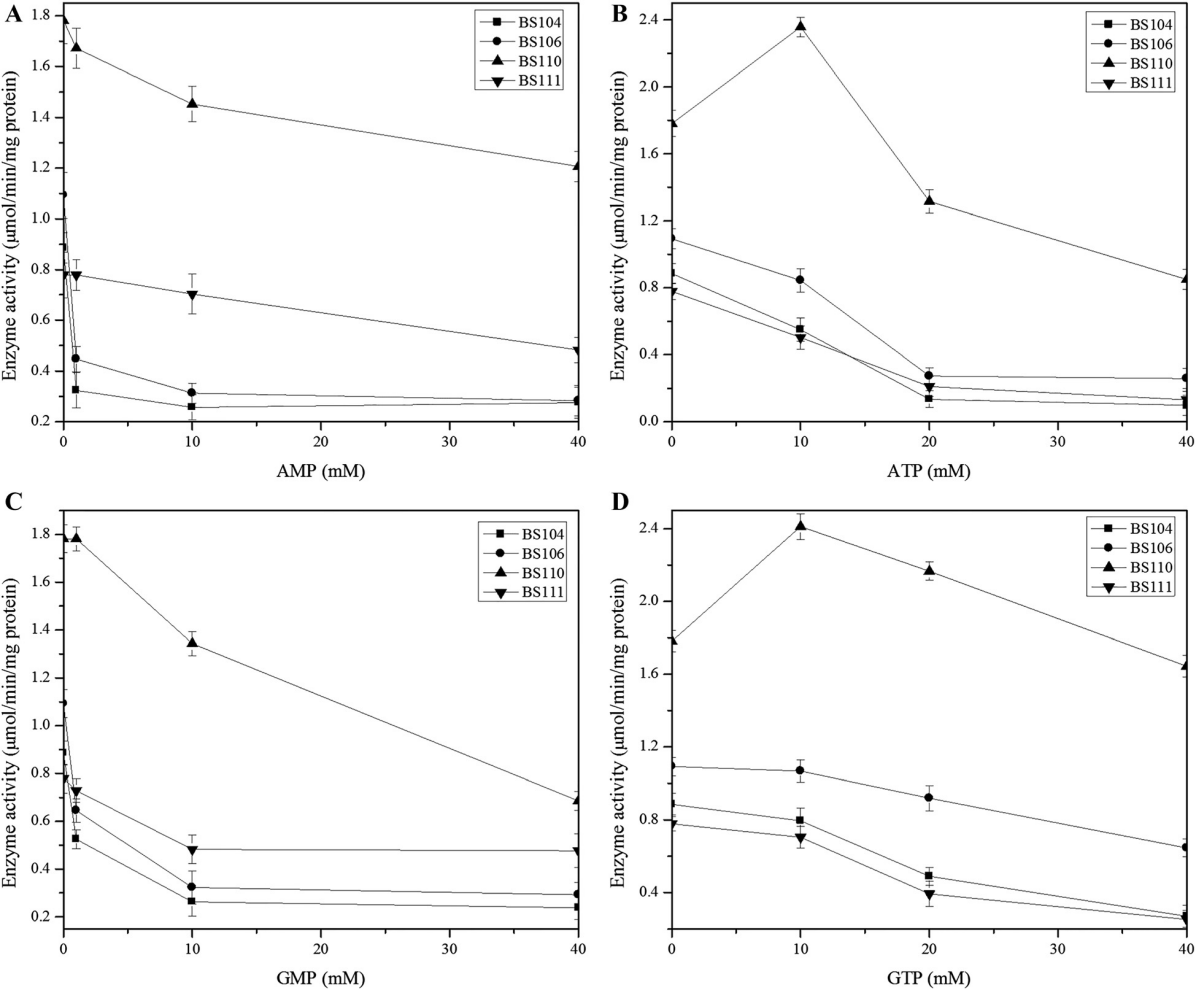
**Inhibitory effect of purine nucleotides on PRPP amidotransferases from*B. subtilis*mutant strains.** The inhibitory effects of increasing concentrations of AMP **(A)**, ATP **(B)**, GMP **(C)** or GTP **(D)** were determined using crude protein extracts from different *purF* manipulated mutant strains of *B. subtilis*: BS104 (■), BS106 (●), BS110 (▲) and BS111 (▼)*.* Results were averages from three independent experiments, and error bars represented the standard errors of the means.

To evaluate the feedback inhibitory properties of PRPP amidotransferase, we assayed the variation trends of *in vitro* enzyme activities from mutant strains under different concentrations of AMP, GMP, ATP and GTP. These four purine nucleotides have been described as the most effective inhibitors of PRPP amidotransferase [[29]]. As a positive control, the activity of PRPP amidotransferase in strain BS104 was clearly inhibited by all four purine nucleotides AMP, GMP, ATP and GTP (Figure 6). Although enzyme activity was actually improved by overexpression of *purF* in BS106, it was still endured intense feedback inhibition under all four purine nucleotides with different concentrations. For the variation trends of enzyme activities in BS111 with overexpression of mutated *purF* from *E. coli*, we could see both ATP and GTP exerted strong inhibition on the activity, but AMP and GMP produced relieved inhibitory effect. We also checked the feedback inhibitory properties of mutated PRPP amidotransferase from strain BS110 under different concentrations of AMP, GMP, ATP and GTP. The results showed that the introduced residue mutations had obvious effects, which displayed strong refractory effect of PRPP amidotransferase activity on four nucleotides-mediated inhibition. Furthermore, it seemed that low concentrations of ATP and GTP induced an activation of PRPP amidotransferase activity. And the results also illustrated that there still existed feedback inhibition with AMP and GMP, although the improved enzyme activities were much higher than that of BS106.

### Intracellular metabolite target analysis confirmed significant deregulation of purine pathway

Intracellular metabolite target analysis was a good option for detecting the genetic modification effects, as observed realities suggested that a change in the activity of single enzyme-often the result of genetic modification, usually produced a larger signal in the metabolite concentrations than in the metabolic fluxes [[30]]. In this study, to gain further insight into the deregulation of purine pathway for riboflavin biosynthesis, major changes of intracellular purine nucleotide concentrations were evaluated in these mutant strains. The metabolite concentrations with significant differences among mutant strains were listed in Table 2.

The intermediates of purine pathway such as IMP, AMP, GMP and GTP were higher in *purR* disruption mutant BS102 compared with parent strain BS89. It might because the defective PurR deregulated purine pathway so as to up-regulate the relative transcription levels of purine genes and then enhanced the intermediate pools. The related purine nucleotides concentrations in BS104 containing *att* deletion based on BS102 had some variation. The intermediates of AMP, ADP and ATP in the branch pathway for ATP formation were slightly decreased, and that of GMP, GDP and GTP had a certain degree of improvement. From these data, it could be concluded that the enhanced supply of intracellular guanine nucleotides was consistent with the enhancement of riboflavin production in mutant strains BS102 and BS104. The significant deregulation of purine pathway at transcriptional level, facilitated by the stepwise gene-targeted manipulations, was supposed to be the major reason for improvement of both intermediate pools and riboflavin biosynthesis flux.

Moreover, major changes of intermediate purine nucleotide concentrations in different engineered *purF* mutant strains were detected. Table 2 showed that overexpression *purF* in BS106 decreased the concentrations of GMP, GDP and GTP but increased that of AMP, ADP and ATP in contrast with BS104. In addition, inserting *purF-VQW* mutations into BS106 leading to BS110 actually enhanced the concentrations of GMP, GDP and GTP compared with BS106, which showed an activation of purine pathway for riboflavin biosynthesis precursor GTP supply.

## Discussion

In this work, to test the deregulation validity of the *de novo* purine biosynthetic pathway for riboflavin overproduction, genetic manipulations of *rib* operon were previously applied to direct the necessary high levels of carbon flow through the riboflavin biosynthetic pathway in *B. subtilis*. We tried three different ways to further deregulate the *rib* operon by overexpression of the key gene (*ribA*), substitution the native promoter *ribP*1 and the RFN regulatory region *ribO* with strong promoter *P*_43_ and disruption of *ribO* in a riboflavin producer *B. subtilis* strain containing *ribC* and *ribO* mutations. It was surprising that such high relative transcription levels of *rib* genes (*ribG* and *ribA*) were achieved by only overexpression of the key gene *ribA* without modification of the native promoter *ribP*1 and the RFN regulatory region. As Humbelin et al [[31]] reported, a further increase in riboflavin titer was observed when a single copy of constitutively expressed *ribA* was introduced into *B. subtilis* strain RB50::[pRF69]_n_::[pRF93]_m_. Similar results enabled us to conclude that the metabolic flux of rate-limiting step catalyzed by *ribA* played an important role in riboflavin biosynthesis. Furthermore, Mironov et al [[32]] reported deletion of the main leader region of *rib* operon encoding FMN-specific sensor RNA in *B. subtilis* affected riboflavin production. In our study, *ribO* deletion seriously affected the transcription levels of *rib* genes (*ribG* and *ribA*) and made riboflavin yield decreased. Our results indicated that some additional FMN-dependent regulation was involved in *rib* operon control, which is, however, still connected with the primary and secondary structure of the leader region.

Genetic manipulations on the *de novo* purine biosynthetic pathway were applied to enhance the transcription levels of purine genes. In this study, *purR* deletion in strain BS102 elevated the relative transcription levels of seven key purine genes involved in purine metabolism. Even the relative transcription levels of *purE* and *purF* were up to 30 times and *purA* and *guaC* were up to 100 times. The Δ*att* mutation based on BS102 was also effective, with the relative transcription levels of *purE* and *purF* were further enhanced and *guaC* and *purA* were declined. The purR deletion and Δ*att* mutation were both advantageous to the deregulation of purine pathway, reflecting that the riboflavin production was increased from 506.45 ± 3.50 mg/L to 627.15 ± 20.61 mg/L. Asahara et al [[13]] performed gene targeted mutagenesis to construct inosine-producing *B. subtilis* with an enhanced *pur* operon expression, which including *purR* and *att* inactivation and the original -10 sequence (TAAGAT) of *Ppur* mutation. The effects of *purR* and *att* inactivation were consistent with our experimental results for the enhanced relative transcription levels of purine genes. The -10 mutation was effective in the increase of inosine, but it presented little effect on enhancement of the riboflavin production in our study.

As a tightly regulated pathway, purine biosynthesis is rigorously regulated at transcription and metabolic levels [[2]]. The committed step of pathway, the formation of PRA, seems to be critical [[33]]. To deregulate the formation of PRA at both transcription and metabolic levels, we firstly inserted a strong promoter *P*_43_ at the *purF* locus in BS104 to generate mutant BS106 for co-overexpression of the *purF*, *purM*, *purN*, *purH*, *purD* genes in *pur* operon. Better performances were exhibited in mutant BS106 with high levels of PRPP amidotransferase activity and improved riboflavin production. But overexpression of *purF* in BS106 was not enough to increase the production of riboflavin up to biotechnologically significant level, because wild-type PRPP amidotransferase was still endured intense feedback inhibition of all four purine nucleotides (AMP, ATP, GMP and GTP) (Figure 6). We then mutated three residues of PRPP amidotransferase that were homologous to those reported to be essential for purine nucleotides binding in *E. coli* [[16],[17]]. Intriguingly, the *purF-VQW* mutations in BS110 led an important increase in riboflavin production, whereas caused growth delay (from 0.60 ± 0.01 h^-1^ to 0.45 ± 0.01 h^-1^). The reason for this discrepancy might be related to the metabolic imbalance of purine pathway caused by PurF disturbance. Moreover, a defective GMP reductase (*guaC*) in BS110 was found to restore the specific growth rate.

With respect to mutant enzyme activity, to the best of our knowledge, it is the first report of *B. subtilis* PRPP amidotransferase with D293V, K316Q and S400W mutations to trigger a strong refractory effect on purine nucleotides mediated inhibition. The mutant PurF enzyme activity from strain BS110 suggested that key mutations in PurF severely affected the affinity of purine nucleotides for their binding sites, abolished the inhibitory properties and released PRPP amidotransferase from the feedback regulation. Surprisingly, by assaying the variation trends of *in vitro* enzyme activities from strain BS110 under different concentrations of four purine nucleotides, the nucleotides ATP and GTP exerted a marked stimulatory effect of PRPP amidotransferase at the lowest concentrations assayed. A possible explanation for this effect could be that ATP and GTP might induce a change in the dimerization status or conformation of the protein, which might contribute to improve stabilization and therefore increased protein activity.

Taken together, we clearly demonstrated that the combination abolishment of regulation in *rib* operon and purine pathway was sufficient to enhance the production of riboflavin. Moreover, based on intracellular metabolites analysis, the intracellular purine nucleotide concentrations were enhanced in the stepwise gene-targeted deregulation mutant strains. These data showed an activation of purine pathway for riboflavin biosynthesis precursor GTP supply and led to three times riboflavin production increase in shaker flask. Concerning further metabolic engineering of purine pathway, several potential targets are of interest and could be available. As shown in Figure 1B, PRPP synthetase, encoded by *prs* gene*,* is subject to feedback inhibition by purine nucleotides, with ADP and GDP being the most effective inhibitors. It has been shown mutated PRS (N120S and L135I) in *B. subtilis* was desensitized to feedback inhibition by ADP and GDP [[14]]. And the positive effect on riboflavin production by overexpression of desensitized feedback inhibition PRS mutants have been presented in *A. gossypii* [[34]]. Therefore overexpression of deregulated PRPP synthetase should be effective for improvement of purine pathway metabolic flux. Besides, the *purA* leaky mutation and *guaB* overexpression may be another useful targets for enhancing riboflavin production in *B. subtilis*. Last but not the least, fine-tuning expression of genes or mutants involved in purine pathway should not be neglected until all three of titer, productivity and yield have been optimized.

## Conclusions

A sequential optimization strategy was applied to deregulate purine pathway of *B. subtilis* to create genetic diversities and to improve riboflavin production. As a conclusion, these rational deregulation mechanisms to eliminate both transcription repression and feedback inhibition were functional on increasing of the metabolite concentrations of purine pathway, and it also meant significance for the yield of products of purine nucleotides or directly stemming from purine pathway in *B. subtilis*.

## Methods

### Bacteria strains, medium and chemicals

The bacterial strains used in this work were listed in Table 3. All *B. subtilis* strains were derived from the wild type *B. subtilis* 168. *E. coli* TOP10 was used for routine transformation and maintenance of plasmids. When required, antibiotics were added to the growth media at the following concentrations: 100 μg/mL ampicillin for *E. coli* selection; 5 μg/mL chloramphenicol for *B. subtilis* selection. 5-fluorouracil (5FU) was purchased from Sigma-Aldrich Corporation (Sigma-Aldrich, St Louis, MO, USA) and prepared as a stock solution of 100 mM in dimethyl sulfoxide (DMSO). The restriction enzymes and T4 ligase were purchased from Thermo Scientific Corporation (Thermo Scientific, Rochester, USA). Taq DNA polymerase was purchased from NEB (New England BioLabs, Ipswich, MA). Antibiotics and other chemicals used in this study were purchased from Sigma-Aldrich Corporation (Sigma-Aldrich, St Louis, MO, USA). All of the chemicals were of reagent grade.

**Table 3:** Bacterial strains and plasmids used in this study

**Name**	**Relevant genotype**^**a**^	**Source/reference**
Strains		
*B. subtilis* 168	Wide-type strain, *trpC2*	Lab Stock
*E. coli* DH5α	Standard cloning strain	Invitrogen
BS77	*B. subtilis* 168Δupp ribC* ribO* yhcF* yvrH* ywaA*	This study
BS89	BS77, *P*_*43*_::*ribA*	This study
BS93	BS77, *P*_*43*_::*ribA P*_*43*_::*ribG* Δ*ribP1* Δ*ribO*	This study
BS96	BS77, *P*_*43*_::*ribA* Δ*ribO*	This study
BS102	BS77, *P*_*43*_::*ribA* Δ*purR*	This study
BS103	BS77, *P*_*43*_::*ribA* Δ*purR P*_*pur*_(-10*)	This study
BS104	BS77, *P*_*43*_::*ribA* Δ*purR* Δ*att*	This study
BS106	BS77, *P*_*43*_::*ribA* Δ*purR* Δ*att P*_*43*_::*purF*	This study
BS107	BS77, *P*_*43*_::*ribA* Δ*purR* Δ*att P*_*pur*_(-10*)	This study
BS110	BS77, *P*_*43*_::*ribA* Δ*purR* Δ*att P*_*43*_::*purF*^D293V K316Q S400W^	This study
BS111	BS77, *P*_*43*_::*ribA* Δ*purR* Δ*att P*_*43*_::*purF*^K326Q P410W(*E. coli*)^	This study
Plasmids		
pUC18	Amp^r^	Lab Stock
pC194	Cm^r^, *Bacillus* cloning vector	Lab Stock
pSS	Amp^r^, Cm^r^, pUC18 containing *cat-upp* cassette	[[36]]
pSS-P_43_-ribA*-*FB	Amp^r^, Cm^r^, containing *ribA* upstream and downstream flanks under *P*_*43*_ promoter	This study
pSS-P_43_-ribG-FB	Amp^r^, Cm^r^, containing *ribG* upstream and downstream flanks under *P*_*43*_ promoter	This study
pSS-ribO*-*FB	Amp^r^, Cm^r^, containing *ribO* flanks	This study
pSS-purR*-*FB	Amp^r^, Cm^r^, containing *purR* flanks	This study
pSS-(-10*)*-*FB	Amp^r^, Cm^r^, containing *Ppur*(-10*) mutant flanks	This study
pSS-att*-*FB	Amp^r^, Cm^r^, containing *att* flanks	This study
pSS-P_43_-purF*-*FB	Amp^r^, Cm^r^, containing *purF* (from *B. subtilis*) upstream and downstream flanks under *P*_*43*_ promoter	This study
pSS-purF*(bsu)*-*FB	Amp^r^, Cm^r^, containing *purF* mutant (from *B. subtilis*) flanks	This study
pSS-purF*(eco)-FB	Amp^r^, Cm^r^, containing *purF* mutant (from *E.coli*) upstream and downstream flanks under *P*_*43*_ promoter	This study

^a^Antibiotic resistance genes in plasmids were abbreviated as follows: Amp^R^, ampicillin resistance; Cm^R^, chloramphenicol resistance.

The minimal medium and shake cultivation medium for physiological characterizations and fermentation procedure were described as previously reported by Wang et al [[35]]. Physiological characterizations were carried out in minimal medium and riboflavin production was carried out in shake cultivation medium. To test the riboflavin biosynthetic activity of mutant strains, single colony was transferred into 5 mL Luria-Bertani (LB) medium and incubated at 41°C in a rotatory shaker at 240 rpm for 14 h to prepare the inocula. 2% (v/v) inocula was added aseptically to a 500 mL shake flask containing 50 mL shake cultivation medium. The fermentation was incubated at 41°C in shake flasks at 240 rpm for 72 h. The LBG medium (LB medium with 1% glucose) was used for quantitative RT-PCR analysis. The M medium was used for intracellular metabolite profile analysis [[35]]. The MM medium containing 10 μm 5FU was used for counter-selection of 5FU resistance colonies [[36]]. All the experiments were carried out independently in biological triplicates, and the reported results were the average of three replicate experiments.

### Plasmids construction

The plasmids used in this study were shown in Table 3. All primers used in their constructions were shown in Additional file 1: Table S1. This vector pSS as described previously by Shi et al [[36]] was used as backbone for all plasmids construction in this study.

For gene in-frame deletion, the upstream and downstream fragments of coding sequence of target gene were amplified by PCR and then inserted into the MCS-I and MCS-II sites of pSS plasmid, respectively. A similar procedure for disruption of *purR*, *att* (the attenuator region in 5’-UTR) and *ribO* was carried out. Taking pSS-purR-FB for example, the *purR* upstream fragment purR-F was amplified from *B. subtilis* genome using primers purR-F-U/L and was digested with *Bgl*II-*Xho*I to ligate into the MCS-I of pSS. Then, the *purR* downstream fragment purR-B was amplified from *B. subtilis* genome using primers purR-B-U/L and was digested with *Sal*I-*Bam*HI to ligate into the MCS-II to generate pSS-purR-FB. The primers purR-F-L and purR-B-F were designed with the complementary sequence as direct repeat sequence (DR) for counter-selectable cassette eviction. Following the same procedure, plasmids pSS-att-FB and pSS-ribO-FB were constructed.

For *purF* overexpression, the *purF* upstream fragment purF-F was firstly amplified from *B. subtilis* genome using primers purF-F-U/L and was digested with *Nde*I-*Xho*I to ligate into the MCS-I of pSS. Second, the strong promoter *P*_*43*_ and *purF* gene were both amplified from *B. subtilis* genome using primers O-P43-U/L and purF-B-U/L, respectively. The primers O-P43-L and purF-B-U were designed as the complementary sequences. These two fragments were then seamlessly joined by splice overlap extension PCR (SOE-PCR) using nested PCR primers Fusion-purF-B-U/L to generate the downstream fragment purF-B, where promoter *P*_43_ was placed upstream of *purF* gene. Then, the downstream fragment purF-B was digested with *Pst*I-*Bam*HI to ligate into the MCS-II to generate pSS-P43-purF-FB for overexpression of *purF*. The DR was designed both at the backward primer purF-F-L of the upstream fragment and the forward primer Fusion-purF-B-U of the downstream fragment. Plasmids pSS-P43-ribA-FB, pSS-P43-ribG-FB and pSS-purF*(eco)-FB were constructed following the same procedure. In pSS-purF*(eco)-FB, the upstream fragment was amplified from strain BS106, and the downstream fragment was created by SOE-PCR containing both mutated *purF* gene from *E. coli* kindly donated by my colleague Dr. Zhenquan Lin and homologous region from strain BS106.

For markerless gene point mutation, the main procedure of plasmid construction was essentially the same as that of gene deletion expect that the point mutations were introduced by primers. Plasmids pSS-(-10)*-FB and pSS-purF*(bsu)-FB were constructed for markerless mutation of the promoter of *pur* operon and *purF* gene of *B. subtilis*. For construction plasmid pSS-(-10)*-FB, the upstream and downstream fragments were amplified by PCR and then inserted into the MCS-I and MCS-II sites of pSS, respectively. It’s worth noting that the point mutations were simultaneously introduced into the backward primer of the upstream fragment and the forward primer of the downstream fragment. For construction plasmid pSS-purF*(bsu)-FB containing three point mutations, the first point mutation (A878T) was introduced by the backward primer of the upstream fragment, the second (A946C) was introduced by the forward nested PCR primer of the downstream fragment, and the third (CC1199GG) was introduced by the primers that were used for the downstream fragment generated by SOE-PCR.

### Strains construction

The construction of mutant strains was based on a mutation delivery system as described previously by Shi et al. [[36]]. The plasmid pSS-P43-ribA-FB was integrated into BS77 chromosome by the first double-crossover chromosomal transformation and the transformant was selected by chloromycetin. Next, the resulting transformant was cultured in LB liquid medium for 12 h, and then the cells were spread on a MM plate containing 10 μM 5FU. The grown colonies on 5FU MM plate were verified by PCR using primers ribA-F-U and ribA-B-L and by Sanger sequencing to confirm that the *upp*-cassette was popped out. After the *upp*-cassette recycling event, the strong promoter *P*_*43*_ was introduced upstream of *ribA* locus to generate the *ribA* overexpression strain, which was denoted as BS89. The *rib* operon in strain BS89 was constitutively expressed by substitution the native promoter *ribP*1 and the RFN regulatory element *ribO* with strong promoter *P*_43_ using plasmid pSS-P43-ribG-FB to generate strain BS93. The RFN regulatory element *ribO* in strain BS89 was knocked out using plasmid pSS-ribO-FB to yield strain BS96.

The plasmid pSS-purR-FB was used to delete *purR* in strain BS89, generating BS102. Plasmids pSS-(-10)*-FB and pSS-att-FB were used to mutate the original -10 sequence and the attenuator region in 5’-UTP of *pur* operon promoter in BS102, individually and in combination, generating strains BS103, BS104 and BS107. Meanwhile, *purF* was overexpressed in strain BS104 to generate strain BS106 using plasmid pSS-P43-purF-FB. Afterwards, *purF* in BS106 was markerlessly mutated using plasmid pSS-purF*(bsu)-FB to generate strain BS110, and was markerlessly substituted by the mutated *purF* gene from *E. coli* using pSS-purF*(eco)-FB to generate BS111.

### Analytical methods

Cell growth was monitored by measuring optical density at 600 nm (OD_600_) with UV-Vis spectrophotometer to monitor the growth of *B. subtilis* strains. Glucose consumption was quantified by a biosensor (SBA-40E, Shandong, China). For riboflavin measurement, samples were first diluted with 0.05 M NaOH and centrifuged at 16,000 × g for 2 min to remove the cells, the supernatant was then diluted by acetic acid sodium*-*acetate buffer solution (pH 5.0) to the linear range of the spectrophotometer and the absorbance at 444 nm was recorded [[35]]. The riboflavin concentration was counted through standard equation which had been validated, Y = (A_444_-0.0057) × DF/0.0321 [R^2^ = 0.9968; Y, the riboflavin concentration of sample (mg/L); A_444_, the value of absorbance at 444 nm; DF, dilution fold; A_444_ was controlled within the range of 0.3-0.8 by dilution].

### Analysis of gene expression by quantitative RT-PCR

Fresh samples of cell cultures, harvested during exponential growth phase in LBG medium, were used to extract total RNA by using RNAprep Pure Cell/Bacteria Kit (Tiangen, Beijing, China) following the manufacturer’s instructions. The cDNA synthesis was prepared using FastQuant RT Kit (with gDNase) (Tiangen, Beijing, China) with random primers. The quantitative RT-PCR was carried out by Light Cycler® 480 II (Roche, Basel, Switzerland) with Real Master Mix (SYBR Green) according to the manufacturer’s instructions as follows. In brief, 100 ng of cDNA was used in a total reaction volume of 20 μl with 0.25 mM of each primer (See Additional file 1: Table S1). The fold change of each transcript in each sample relative to the control was measured in triplicates, normalized to internal control gene *rrna* and calculated according to the comparative *C*_T_ method [[37]].

### Enzyme activity assays

The crude cell extracts were prepared as described previously by Shi et al [[23]]. PRPP amidotransferase activity was assayed by measurement of the transformation from glutamine into glutamic acid according to the following reaction: Glutamine + PRPP → PRA + Glutamic acid. The standard assay for total PRPP amidotransferase was performed as described previously by Kim et al [[38]]. The PRPP amidotransferase activity was expressed as nanomoles glutamic acid formed per microgram of protein extract per minute. Total protein concentrations were determined according to the Bradford method [[39]].

### Intracellular metabolite target analysis

For sampling, 10 mL of cell suspension was harvested during exponential growth phase in M medium using vacuum filtration (-40 to -50 kPa, AP-01P Vacuum Pump, Tianjin Auto Science Co., Ltd.; cellulose nitrate, 0.45 μm pore size. The whole filtration procedure including the washing was completed in less than 30 s.) and washed twice with 2 mL of NaCl solution (2.6%, 20-25°C, the whole filtration procedure including the washing was completed in less than 30 s) [[40]]. Cells harvested were incubated in 2 mL of -20°C 40:40:20 acetonitrile/methanol/water for metabolite extraction in parallel. Next, the extracts were cooled on ice, transferred into 2 mL tubes, and centrifuged (10 min, 16,000 g, 4°C, PM180R, Italy) to remove cell debris. Then, all samples were lyophilized and redissolved in 200 μL 40:40:20 acetonitrile/methanol/water for further metabolite analysis.

Measurement of intracellular purine metabolites was carried out by liquid chromatography-tandem quadrupole mass spectrometry (LC-MS/MS). Metabolites were firstly separated by aminopropyl column on a Phenomenex Luna NH_2_ (250 × 4.6 mm, 5 μm) at a flow rate of 1 mL/min. In all cases, 10 μL standard solution or sample was injected. The mobile phase consisted of solvent A (20 mM ammonium acetate + 20 mM ammonium hydroxide in 95:5 water/acetonitrile, pH 9.45) and solvent B (acetonitrile), the metabolites were eluted using a linear gradient of solvent A 98-40% from 0 to 10 min, 40-24% from 10 to 20 min [[41]]. The metabolites mass spectrometric data were then collected with a Bruker microOTOF-Q II mass spectrometer operated in negative ionization electrospray mode. Compounds were identified in cell extracts using Metabolite Tools™ software and the parameters of compounds, such as retention time, m/z, peak areas, and specific fragmentation pattern, were also identified. Thus, the concentrations of intracellular purine metabolites were quantified based on their standard curves, which were established by plotting the peak areas vs concentrations of each standard solution.

## Competing interests

The authors declare that they have no competing interests.

## Authors’ contributions

TS and YW performed the experiments under the guidance of ZW and XZ. TS, YW and ZW analyzed the experimental data and drafted the manuscript. TC, DL and JF made substantial contributions to conception, interpretation of data and revised the manuscript. ZW, TS and GW developed the idea for the study and designed the research. All authors read and approved the final manuscript.

## Additional file

## Supplementary Material

Additional file 1: Table S1. Primers used in this study. Click here for file
